# Epstein-Barr Virus-Related Infectious Mononucleosis Accompanied by Cholestatic Hepatitis

**DOI:** 10.7759/cureus.88392

**Published:** 2025-07-20

**Authors:** Hiroaki Nishioka, Nanako Kitagawa

**Affiliations:** 1 Department of General Internal Medicine, Kobe City Medical Center General Hospital, Kobe, JPN

**Keywords:** cholestatic hepatitis, epstein-barr virus, infectious mononucleosis (im), jaundice, subserosal gallbladder edema

## Abstract

Infectious mononucleosis, caused by Epstein-Barr virus (EBV), is a common infection in young adults. It typically presents with a self-limiting course characterized by fevers, pharyngitis, and lymphadenopathy. While mildly elevated liver enzymes are a common complication, cholestatic hepatitis and clinical jaundice are rare in EBV infection. This report describes the case of a young woman who developed EBV-related infectious mononucleosis, accompanied by cholestatic hepatitis and jaundice. The patient presented with fever, sore throat, and whitish, loose stools. She exhibited yellowing of ocular conjunctivae and skin, enlarged tonsils with white patches, and palpable liver. Laboratory findings showed leukocytosis with atypical lymphocytes, hyperbilirubinemia, and significantly elevated liver enzymes. A computed tomography scan revealed hepatosplenomegaly and subserosal gallbladder edema. The patient was diagnosed with infectious mononucleosis, complicated with cholestatic hepatitis and jaundice. Serologic testing confirmed the presence of EBV infection. Her condition and laboratory results improved without specific treatment. Infectious mononucleosis due to EBV, though rarely presenting with jaundice, should be considered in young individuals with fever and sore throat to avoid misdiagnosis and unnecessary investigations.

## Introduction

Epstein-Barr virus (EBV) is a member of the herpes virus family and is highly prevalent in the general population [1]. While primary EBV infection in children is usually asymptomatic, it can lead to a clinical syndrome known as infectious mononucleosis, particularly in adolescents and young adults. Common symptoms include fatigue, fever, sore throat, and enlarged lymph nodes [2]. Liver involvement may occur, typically resulting in mildly and temporarily elevated liver enzymes [3].

However, only 5% or fewer of EBV infections present with jaundice, and even fewer cases are caused by cholestatic hepatitis [4,5], making this a rare but clinically significant manifestation.

Edematous gallbladder wall thickening, due to venous congestion, presents with subserosal edema and lacks inflammatory signs, distinguishing it from infectious causes, such as cholecystitis, which show mural hyperenhancement and pericholecystic fluid. While infectious wall thickening is often associated with gallbladder distension and diffuse wall thickening, edematous wall thickening on computed tomography scans is characterized by subserosal edema and the periportal collar sign, both of which reflect venous blood congestion [6]. In EBV-related infectious mononucleosis, cases of edematous wall thickening in the gallbladder are rarely reported.

In this report, we present the case of a young woman who developed EBV-related infectious mononucleosis accompanied by cholestatic hepatitis, which manifested as jaundice and edematous wall thickening of the gallbladder.

## Case presentation

A previously healthy 25-year-old woman presented with a 10-day history of fever and sore throat, and a three-day history of whitish, loose stools. Physical examination revealed yellowing of ocular conjunctivae and skin, enlarged tonsils with white patches, and palpable liver. Laboratory findings included: white blood cell count, 10,800/µL, with 22.5% atypical lymphocytes; hemoglobin, 13.2 g/dL; total bilirubin, 5.1 mg/dL; direct bilirubin, 4.0 mg/dL; aspartate aminotransferase, 550 U/L; alanine aminotransferase, 1033 U/L; alkaline phosphatase (ALP), 794 U/L; γ-glutamyl transpeptidase (γ-GTP), 453 U/L (Table 1).

**Table 1:** Blood test results on admission T-BIL: total bilirubin, D-BIL: direct bilirubin, AST: aspartate aminotransferase, ALT: alanine aminotransferase, ALP: alkaline phosphatase, γ-GTP: γ-glutamyl transpeptidase, BUN: blood urea nitrogen, Cre: creatinine, CRP: C-reactive protein.

Test items	Result	Reference range
White blood cell (/μL)	10,800	3900-9800
Neutrophils (%)	36.5	26-71
Lymphocyte (%)	38.5	19-61
Atypical lymphocyte (%)	22.5	0.0-0.0
Hemoglobin (g/dL)	13.2	11.1-15.1
Platelet (×10^4^/μL)	21.4	13.0-37.0
T-BIL (mg/dL)	5.1	0.2-1.2
D-BIL (mg/dL)	4.0	0.1-0.7
AST (U/L)	550	8-40
ALT (U/L)	1033	8-40
ALP (U/L)	794	38-113
γ-GTP (U/L)	453	0-69
BUN (mg/dL)	6.8	8.0-20.0
Cre (mg/dL)	0.7	0.40-1.80
CRP (mg/dL)	1.64	0.00-0.50

**Figure 1:**
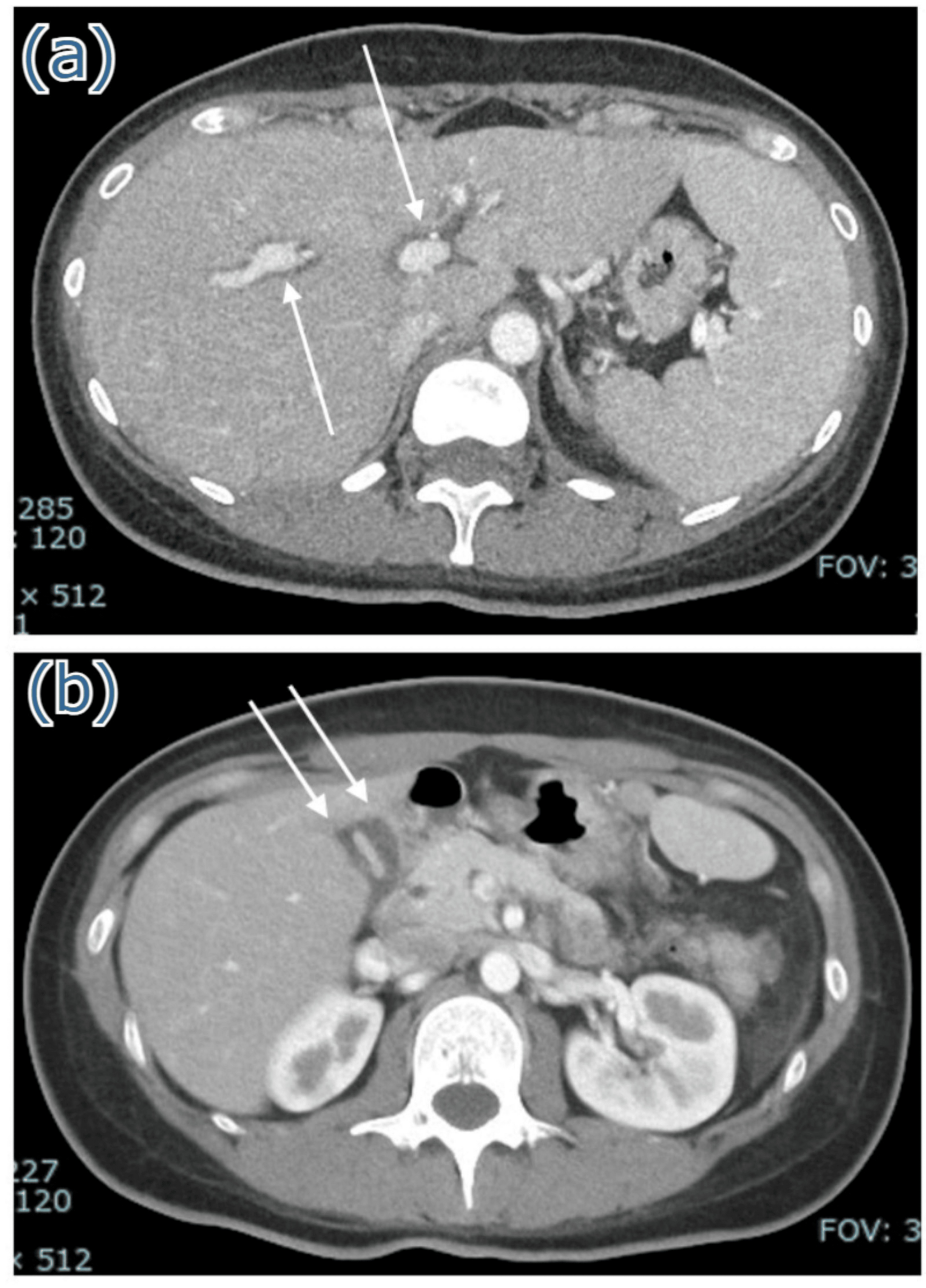
Computed tomography images. Abdominal contrast-enhanced computed tomography showing (a) hepatosplenomegaly and periportal collar sign in the liver (white arrows), and (b) subserosal edema of the gallbladder (white arrows).

Serologic results showing positive viral capsid antigen-IgG with negative EB nuclear antigen (EBNA)-IgG were consistent with a primary acute EBV infection. Cytomegalovirus IgG and IgM antibodies were negative. Contrast-enhanced computed tomography (CT) scan revealed hepatosplenomegaly, periportal collar sign in the liver, and subserosal gallbladder edema (Figures 1a, 1b).

The patient was diagnosed with infectious mononucleosis, accompanied by cholestatic hepatitis and jaundice. Her symptoms and liver function gradually improved with supportive care, without the need for antiviral or steroid treatment. Two months later, the EBNA IgG test result returned positive, confirming that the infectious mononucleosis was due to EBV infection. 

## Discussion

The clinical course of our patient highlighted two important observations. First, EBV-related infectious mononucleosis can cause cholestatic hepatitis with clinical jaundice. Second, edematous wall thickening of the gallbladder can be observed in EBV-related infectious mononucleosis.

Infectious mononucleosis is a common infection in adolescents and young adults, mainly caused by EBV. Most cases are self-limited. Typical symptoms include fever, sore throat, fatigue, and lymphadenopathy [2]. While mild elevations of liver enzymes are common in patients with infectious mononucleosis, a handful of cases of cholestatic hepatitis with significant jaundice have been reported [4-9]. In the current case, the predominance of direct hyperbilirubinemia, along with significant elevations in hepatobiliary enzymes, indicated a mixed liver injury consistent with cholestatic hepatitis. The condition typically presents with elevated direct bilirubin levels, along with ALP and γ-GTP levels that are at least three times the upper limit of normal. Additionally, aminotransferase values can be up to seven times higher than the upper limit of normal [10]. The precise mechanism of liver cell damage leading to cholestasis remains unclear, as EBV does not cause direct cytotoxic effects on hepatocytes. Cholestasis in EBV infection is hypothesized to result from oxidative stress, involving lipid peroxidation and free radical accumulation, which may disrupt bile canalicular transport [11,12].

Jaundice in EBV infections is rare and occurs in less than 5% of patients [4,5]. It can result from various issues, including autoimmune hemolytic anemia, cholestasis due to acalculous cholecystitis, and biliary duct obstruction caused by abdominal lymphadenopathy [13-15]. Our patient showed no signs of anemia and had direct bilirubinemia, making hemolytic anemia an unlikely cause of jaundice. An abdominal CT scan ruled out extrahepatic cholestasis.

Edematous wall thickening of the gallbladder, as seen in this case, can develop due to venous blood congestion and is commonly associated with conditions such as heart failure, hypoproteinemia, liver cirrhosis, and acute hepatitis [5]. Our patient was a healthy young woman, and no cause other than acute cholestatic hepatitis was identified based on her medical history, physical examination, blood test results, and imaging findings. On abdominal CT scans, this thickening is characterized by subserosal edema, largely because the connective tissue surrounding the gallbladder is thin and dense. Additionally, it is commonly accompanied by periportal collar sign, indicating the presence of periportal edema [6]. Distinguishing edematous wall thickening from infectious wall thickening, which can occur in cases of acute cholecystitis, is clinically important, because the treatments are different. While acute cholecystitis generally requires aggressive treatment such as antimicrobial administration, drainage, and cholecystectomy, edematous wall thickening in infectious mononucleosis may not require specific treatment. Although acalculous cholecystitis has been described in cases of EBV-related infectious mononucleosis [15], gallbladder wall thickening due to edema, rather than inflammation, is a rare finding, which may avoid unnecessary surgical or antibiotic intervention.

## Conclusions

This case demonstrates that EBV-related infectious mononucleosis can lead to cholestatic hepatitis, which may result in jaundice and present with edematous thickening of the gallbladder wall. The present case emphasizes the need to consider infectious mononucleosis caused by EBV as a potential diagnosis in young individuals who show fever, sore throat, and jaundice, especially in the absence of anemia or biliary obstruction. Early recognition of EBV infection can help prevent unnecessary diagnostic tests and enable timely and appropriate management.
